# What is a “sense of foreshortened future?” A phenomenological study of trauma, trust, and time

**DOI:** 10.3389/fpsyg.2014.01026

**Published:** 2014-09-17

**Authors:** Matthew Ratcliffe, Mark Ruddell, Benedict Smith

**Affiliations:** ^1^Department of Philosophy, Durham UniversityDurham, UK; ^2^Northumberland, Tyne and Wear NHS Foundation TrustNewcastle upon Tyne, UK

**Keywords:** anticipation, Husserl, interpersonal relations, temporal experience, torture, trauma, trust

## Abstract

One of the symptoms of trauma is said to be a “sense of foreshortened future.” Without further qualification, it is not clear how to interpret this. In this paper, we offer a phenomenological account of what the experience consists of. To do so, we focus on the effects of torture. We describe how traumatic events, especially those that are deliberately inflicted by other people, can lead to a loss of “trust” or “confidence” in the world. This undermines the intelligibility of one’s projects, cares, and commitments, in a way that amounts to a change in the structure of temporal experience. The paper concludes by briefly addressing the implications of this for how we respond to trauma, as well as offering some remarks on the relationship between trauma and psychosis.

## INTRODUCTION

The Istanbul Protocol, a United Nations guide for investigating and documenting cases of torture, describes one of its long-term effects as follows:

The victim has a subjective feeling of having been irreparably damaged and having undergone an irreversible personality change. He or she has a sense of foreshortened future without expectation of a career, marriage, children, or normal lifespan (Istanbul Protocol, 1999, p. 47).

A “sense of foreshortened future” is also mentioned as a symptom of post-traumatic stress disorder (PTSD) in the fourth edition of the *Diagnostic and Statistical Manual of Mental Disorders* (DSM-IV-TR, p. 468). In DSM-5, there is a more general statement about various negative expectations concerning the future. Here, we focus more specifically on the “foreshortened future” criterion. What might the relevant experience consist of? The DSM and Istanbul Protocol do not provide any further clarification, and the cursory descriptions that they do offer are compatible with various interpretations. On one account, a sense of foreshortened future involves a cluster of interrelated judgments regarding what the future most likely holds, such as “I will die young,” “I will not have a family,” and “I will not have a successful career.” These either comprise or originate in a more general evaluation of future events, the content of which is something like “bad things are going to happen to me and good things are not going to happen to me.” In conjunction with this, negative emotions toward the future predominate: one fears that *p* and *q*, rather than hoping that *r* and *s*. If this is right, then a sense of foreshortened future is to be distinguished from an experience of *time* itself or – if you prefer – from an experience of *temporal properties*. The traumatized person continues to distinguish past, present, and future, to anticipate the arrival of future events, and to experience temporal passage. Regardless of whether she anticipates future event *p*, where *p* is evaluated negatively (most notably, her own premature death), or future event *q*, where *q* is evaluated positively, both are experienced as “future” in the same way. So a foreshortened future is a matter of *what* is anticipated, a negative evaluation of what the future offers rather than an altered sense of the future itself.

In contrast to that view, we will argue that the experience of time is itself affected. Rather than a change in what is anticipated, arising against a backdrop of intact temporal experience, there is an altered sense of temporal passage, of “moving forward” in time, along with a change in how past, present, future, and the relationship between them are experienced. Certain other symptoms of trauma, such as “flashbacks” and feeling unable to “move on” from what has happened, can also be understood in terms of this. In what follows, we provide a phenomenological account, one that serves to illuminate the nature of a poorly understood change in the overall structure of experience that sometimes (but not always) follows exposure to certain events.

## TORTURE AND TRAUMA

The experience addressed here is documented both as an effect of torture and as a symptom of PTSD. The focus of our discussion therefore needs to be clearer. Definitions of torture are contested. According to the Istanbul Protocol, it involves inflicting mental or physical suffering in order to punish, intimidate, obtain information, or extract a confession, and a public official must be implicated^1^. Others reject the “public official” condition as too restrictive. For instance, Kenny (2010) instead emphasizes the “instrumentality” of torture: suffering is intentionally caused in order to elicit a behavioral response, in a situation where the torturer has control over the victim^2^. The two psychiatric diagnoses most often associated with the effects of torture are major depression and PTSD, which are difficult to tease apart when diagnosed together (Istanbul Protocol, 1999, p. 45). As it is debatable what “torture” consists of and the kind of experience we describe is not unique to torture victims, it might seem preferable to widen the scope of our enquiry to PTSD and depression. However, the diagnostic category PTSD admits considerable heterogeneity, and there are also high levels of comorbidity with several other psychiatric conditions (DSM-IV, p. 465). The same points apply to the category “major depression” (Ratcliffe, in press). Furthermore, some experiences of trauma, such as “war trauma,” involve a wider range of symptoms than those associated with PTSD and/or major depression (Ford, 1999; Hunt, 2010). Matters are further complicated by the overlap between grief and trauma, given that grief sometimes involves bereavement by “traumatic means” (Neria and Litz, 2004, p. 75). So it is unlikely that one or another psychiatric category will be reliably associated with a distinctive kind of experience.

The experience we seek to characterize might be associated with a diagnosis of PTSD, major depression or both, but is not a prerequisite for either. It is better captured by the ICD-10 subcategory of “enduring personality change after catastrophic experience,” the symptoms of which include “a hostile or mistrustful attitude toward the world,” “social withdrawal,” “feelings of emptiness of hopelessness,” “a chronic feeling of being ‘on the edge’, as if constantly threatened,” and “estrangement” (ICD-10, p. 209). And it is also consistent with Judith Herman’s account of what she calls “complex PTSD” or “disorders of extreme stress not otherwise specified” (Herman, 1992/1997; Ford, 1999). However, given that (a) the experience is not specific to any one psychiatric diagnosis, (b) many of the relevant diagnostic categories are contested, and (c) all of these categories are also compatible with other – often subtly different – kinds of experience, we do not tie our subject matter to one or another diagnosis. Instead, we focus on a certain kind of traumatic event, one where extreme suffering is deliberately inflicted upon a person by others. This is a necessary but not – according to most accounts –sufficient condition for torture. Our discussion therefore applies equally to the potential psychological effects of sexual assault, domestic violence, prejudicial discrimination, enforced periods of extended solitary confinement, and other forms of physical and psychological abuse, regardless of whether or not they are classified as forms of torture.

In referring to “trauma” it is debatable whether the emphasis should be on events that are intrinsically traumatic according to some criterion or whether any event that affects a given person in the relevant way qualifies as “traumatic” in that instance. However, our emphasis is on occurrences that would cause almost anyone great distress, and so the distinction between traumatic events and the traumatic effects of events is not so pressing^3^. We thus refer to “traumatic events” and also to enduring “traumatic experiences” or “experiences of trauma” that follow them. Of course, people respond in different ways to traumatic events, due to factors that include age, gender, idiosyncratic dispositions, life history, interpersonal relationships, and how events are interpreted and re-interpreted individually and culturally. Furthermore, effects of traumatic events can be hard to distinguish from effects of wider social and cultural upheavals, as is often the case in refugee populations. The potentially different effects of discrete traumatic events and repeated or chronic exposure should also be kept in mind (Herman, 1992/1997; Ford, 1999)^4^.

So the kind of experience addressed here does not inevitably follow interpersonal trauma and it is not exclusive to interpersonal trauma. Nevertheless, there is something distinctive about the psychological effects of harm inflicted by others. As Janoff-Bulman (1992, p. 77) observes, being “singled out for injury […] by another person […] presents particular challenges to the victim’s assumptive world.” We consider the nature of these “challenges” to one’s “assumptions.” We will first describe a pervasive shift in how the person relates to others that can follow interpersonal trauma, something that is often described as a “loss of trust.” We will suggest that this centrally involves a pervasive alteration in how events are anticipated, which – in the most extreme cases – renders a purposive orientation toward a meaningful future unintelligible to the person. This, we will further show, amounts to a profound shift in the experience of time. As indicated by the emphasis upon “loss” of trust, an important limitation of our analysis is that it concerns cases where a previously intact (or largely intact) sense of trust is disturbed by traumatic events. Hence we do not address the various ways in which and degrees to which trust might be disrupted and developmentally derailed before it is fully formed, as happens when traumatic events occur during childhood. Although these need to be considered separately, our account of what the relevant kind of “trust” comprises will be applicable here too.

## LOSS OF TRUST

A sense that the future is bereft of positive, meaningful life events is equally a sense that one’s meaningful life is in the past, finished. So remarks to the effect that the future has nothing to offer are sometimes accompanied by the claim that one has died, that part of one has died, or that one persists but no longer “lives:” “I felt as though I’d somehow outlived myself” (Brison, 2002, p. 9). This corresponds to a wider phenomenon that Freeman (2000, p. 90) has called “narrative foreclosure,” defined as “the premature conviction that one’s life story has effectively ended: there is no more to tell; there is no more than *can* be told.” It is not simply that the person believes she does not have much time left; the traumatic event somehow disrupts her ongoing life story such that the story ceases to be sustainable. (A “life story,” for current purposes, is a meaningful, coherent interpretation of past activities, relationships, achievements, and failures, which also includes a sense of where one is heading – what one’s cares, commitments, and projects currently consist of, and what one seeks to achieve.) Even if something like this is right – and we think it is – it does not tell us *why* a life story has collapsed. Let us consider three scenarios:

(1) Loss of a life narrative is constitutive of a sense of foreshortened future.
(2) Loss of a life narrative is symptomatic of a loss of projects, cares, and commitments upon which that narrative is founded.
(3) Both (1) and (2) are symptomatic of losing something that is presupposed by the intelligibility of life narratives and life projects.

We doubt that a sharp distinction can be drawn between (1) and (2), given that the ability to develop, sustain, and revise projects and commitments plausibly incorporates the ability to situate them within a cohesive, purposive, forward-looking narrative, one that is told and retold to oneself and others. We do not wish to deny that some instances of narrative foreclosure are attributable largely or wholly to a combination of (1) and (2). Life projects and commitments depend upon a wider social and cultural environment, and are therefore vulnerable to collapse in the event of certain changes in that environment. For example, if universities ceased to exist after some major social upheaval, a life focused around excelling as a University Professor would be unsustainable. Numerous projects would collapse, along with a broader, purposive narrative into which they are integrated; a life story would be cut short. Lear (2006) describes how cultural changes can lead to loss of meaning structures that the life-stories and purposive activities of whole communities depend upon. This amounts to a form of narrative foreclosure, a sense that meaningful events can no longer transpire, that nothing can “happen.” However, it is to be distinguished from a superficially similar but importantly different kind of experience. Interpersonal trauma can lead to narrative foreclosure even when the relevant social and cultural structures remain largely intact. Indeed, re-establishing a sense of connection with these structures has been identified as an important goal of rehabilitation (Herman, 1992/1997). Furthermore, trauma is often experienced as specific to oneself; it is something that “I” and “I alone” – rather than “we” – have endured and continue to endure. In such cases, it involves an isolation from others that distinguishes it from shared meaning loss (although the distinction is certainly not clear-cut, and the kind of experience we describe can also involve shared meaning loss). In at least *some* such cases, we will argue, what is lost is not just (1) and/or (2) but also (3). In the type of case Lear describes, an open and meaningful future remains; what is lacking is a more determinate sense of which meaningful possibilities that future includes. However, for some, even this much is lost. There is an alteration in *how* time is experienced, such that the possibility of “moving on” in any kind of purposive, meaningful way can no longer be entertained. We will describe this by first turning to the theme of “trust.”

First-person accounts of severe trauma, especially interpersonal trauma, tend to emphasize a “loss of trust.” There are references to this in diagnostic manuals as well. For instance, DSM-5 (p. 271) refers to “exaggerated negative beliefs” such as “no one can be trusted” and “the world is completely dangerous.” But what, in this context, is “trust?” Trust is often construed by philosophers as a three-place relation, of the form “*x* trusts *y* to do *z*.” Hence the philosophical task becomes that of identifying criteria which distinguish trusting *y* to do *z* from various other attitudes, such as hoping that *y* will do *z* and thinking that *y* will probably do *z*^5^. However, this does not exhaust the scope of “trust.” Three-place trust can be distinguished from two-place trust, where “*x* trusts *y*” full stop, without reference to a specific situation or action. And there is also what we might call “one-place trust,” where one trusts other people in general rather than trusting a specific individual or group of individuals: one “trusts y to do *z*” because one “trusts *y*,” and one trusts *y* because one simply “trusts.” The latter might also be described as “having trust” and is thus analogous to “having hope,” something that need not relate to a specific hope content.

One-place trust is seldom remarked upon in mundane, everyday discourse. However, its loss is a conspicuous theme in first-person accounts of traumatic experience. It might be objected that the term “trust” is misleading here, as “trust” ordinarily refers to the three-place and perhaps also the two-place relation, both of which are quite different. But one-place trust is closely related to the others: one must first *have trust* in order to trust *y* to do *z* or to trust *y* more generally. On the other hand, that *p* is a condition of possibility for *q* does not imply that *p* and *q* are of the same kind. And nothing really hinges on whether we insist on the word “trust.” One reason for retaining it is that people generally *do* refer to what we describe as a form of “trust.” However, a host of related terms are also at play. Jones (2004) calls it “basal security,” while Herman (1992/1997) refers to “basic trust” but also to a sense of “safety in the world.” Améry (1999, p. 47) describes an enduring loss of “trust in the world” that he experienced after torture and subsequent incarceration in Auschwitz, but also emphasizes the broader theme of “security,” which includes an “entire field” of related words, such as “loyal, familiar, confidence, to trust, to entrust^6^.”

“Having trust” might be construed as a non-phenomenological disposition to adopt certain attitudes and have certain kinds of experience. But it also has a phenomenology in its own right; “losing trust” involves losing a habitual confidence that more usually permeates all experience, thought, and activity. It is sometimes described in terms of finding oneself in a different world, a world where people in general seem somehow different: “the entire world of people becomes suspect” (Janoff-Bulman, 1992, p. 79)^7^. Traumatic events are often said to “shatter” a way of experiencing the world and other people that was previously taken for granted:

…we experience a fundamental assault on our right to live, on our personal sense of worth, and further, on our sense that the world (including people) basically supports human life. Our relationship with existence itself is shattered. Existence in this sense includes all the meaning structures that tell us we are a valued and viable part of the fabric of life (Greening, 1990, p. 323).

What, exactly, does this “shattering” involve? It could be that experiencing significant suffering at the hands of another person leads to a negation of engrained beliefs such as “people do not hurt each other for the sake of causing pain,” “people will help me if I am suffering,” and so on. Then again, through our constant exposure to news stories and other sources, most of us are well aware that people seriously harm each other in all manner of ways. One option is to maintain that we do not truly “believe” such things until we endure them ourselves, and various references to loss of trust as the overturning of deeply held “assumptions” lend themselves to that view. For example, Herman (1992/1997, p. 51) states that “traumatic events destroy the victim’s fundamental assumptions about the safety of the world,” and Brison (2002, p. 26) describes how interpersonal trauma “undermined my most fundamental assumptions about the world.” An explicitly cognitive approach, which construes these assumptions as “cognitive schemas” or fundamental beliefs, is adopted by Janoff-Bulman (1992, pp. 5–6), who identifies three such beliefs as central to one-place trust: “the world is benevolent;” “the world is meaningful;” and “the self is worthy.”

However, it seems implausible to insist that “*x* thinks that *x* believes that *p*” when *x* actually believes not *p* until *x* is tortured and really does come to believe that *p*. It could be argued that the term “belief” encompasses more than one type of attitude. So one *a*-believes that *p* but later comes to *b*-believe it too^8^. Another option is to note that the belief “people have done *p* to other people and will do so again” is distinct from “someone might do *p* to a particular person, me.” Hence the deep-rooted belief that is overturned is of the form “*p* won’t (or even can’t) happen to me.” One, the other or both might well be right, but it would be a mistake to construe loss of one-place trust solely in these terms. Although it clearly does involve changes in attitude toward oneself, other people and the world more generally, it also involves something more than this^9^. What is eroded, we suggest, is a habitual, non-propositional *style of anticipation*, which cannot be exhaustively characterized in terms of however many propositional attitudes of the form “*x* believes that *p*.” It is something that a person could describe in terms of the negation of any number of different propositions, including “the world is safe,” “others will help me when I am in trouble,” “people generally mean well,” “good things will happen in the future,” “I will live a long time,” “there are worthwhile projects,” “I am worthy,” and “I have friends.” However, such utterances do not just convey distinct, thematically related belief contents. They are also used to express a unitary and more enveloping phenomenological change, the nature of which can be made clearer by emphasizing the theme of unpredictability:

Massive deconstruction of the absolutisms of everyday life exposes the inescapable contingency of existence on a universe that is random and unpredictable and in which no safety or continuity of being can be assured (Stolorow, 2007, p. 16).

Many of us anticipate most things with habitual confidence. It does not occur to us that we will be deliberately struck by a car as we walk to the shop to buy milk or that we will be assaulted by the stranger we sit next to on a train. There is a sense of security so engrained that we are oblivious to it. Indeed, the more at home we are in the world, the less aware we are that “feeling at home in the world” is even part of our experience (Baier, 1986; Bernstein, 2011). It is not itself an object of experience but something that operates as a backdrop to our perceiving that *p*, thinking that *q* or acting in order to achieve *r*. To lose it is not just to endorse one set of evaluative judgments over another. It is more akin to losses of *practical* confidence that all of us feel on occasion, in relation to one or another performance. Suppose, for instance, one starts to “feel” that one can no longer teach well. Granted, evaluative judgments have a role to play, but loss of confidence need not originate in explicit judgments about one’s performance, and its nature is not exhausted by however many judgments. The lecture theater *looks* somehow different – daunting, oppressive, unpredictable, uncontrollable. Along with this, one’s actions lack their more usual fluidity and one’s words their spontaneity. The experience is centrally one of *feeling* unable to engage in a habitual, practical performance. And loss of confidence can remain resistant to change even when one explicitly endorses propositions such as “I am a good teacher.”

Such an experience can be fairly circumscribed, relating primarily to certain situations. However, we suggest that human experience also has a more enveloping “overall style” of anticipation. This view is developed in some depth by the phenomenologist Husserl (1991). According to Husserl, all of our experiences and activities incorporate anticipation. He uses the term “protention” to refer to an anticipatory structure that is integral to our sense of the present. It is not “added on” to an independently constituted sense of what is present; our experience of an entity *as present* includes anticipation. Husserl adds that a sense of the immediate past is likewise inseparable from the present. When something happens, we do not experience it as “present,” after which it is “gone” or somehow “fades.” Experience includes “retentions,” present experiences of events *as* having just past. The experienced “flow” or “passage” of time involves a structured interplay between protention and retention. An oft used example is that of listening to a melody, where how one experiences a present note is inseparable from a sense of what preceded it, of where it has “come from,” as well as from some sense of what is coming next.

The content of what is anticipated varies in its determinacy, and Husserl (1948/1973) refers to this as “open uncertainty.” For example, as one turns an object over, one might anticipate that its hidden surface will be smooth and red, or one might just anticipate a surface with some color and texture. He contrasts this kind of uncertainty with “problematic uncertainty,” which takes the form “*p* might not occur” or “what looks like *p* may not be *p*,” regardless of how determinate *p* might be. There is also “doubt,” which involves conflict between an earlier anticipation of *p* and a later, competing anticipation of “*q* and not *p*.” Husserl adds that localized experiences of problematic uncertainty and doubt arise against an enduring backdrop of habitual certainty. You might doubt that something is what it seems, as when you look into the distance from a station platform, take a far-away light to be that of your approaching train, and then start to wonder whether it might be something else. But here your experience of doubt is localized. You continue to take it as given that your foot will land on a flat surface as you take a step forward, that the coffee you are about to sip will be warm, that others on the platform will behave in the usual way, and so forth. For the most part, such expectations do not take the form of explicit judgments. They are symptomatic of a habitual, practical confidence, a feeling of being more generally “at home” in the world. It is against this backdrop that we have more localized experiences of problematic uncertainty and doubt, and make explicit judgments to the effect that event *p* will, will not, or might not arise. Hence a non-localized sense of confidence or certainty is not itself an attitude toward anything specific but something that is already in place when such attitudes are adopted^10^.

We can thus distinguish (a) the content of what is anticipated, which may be more or less determinate, from (b) the mode of anticipation, which may be doubt or uncertainty but is more usually certainty. We can add to Husserl’s account (c) the affective “style” of anticipation. When we anticipate events that matter to us in one or another way, we may do so with excitement, hope, curiosity, fear, panic, or dread. Habitual certainty has its own affective style: there is confident engagement with what is coming next, of a kind that is incompatible with fearing or dreading it. And, when we dread *p* (regardless of whether *p* is anticipated in the mode of certainty, problematic uncertainty, or doubt), we generally do so within a wider context of practical confidence, in relation to which *p* is experienced as a localized anomaly.

Were this style of anticipation to break down completely, we could not anticipate localized conflicts in the modes of problematic uncertainty or doubt, given that things appear potentially or actually anomalous in these ways insofar as they are at odds with a wider framework of coherent anticipation. Hence the result would be a loss of experiential structure. What, though, if it were altered in some distinctive way, rather than altogether lost? This, we propose, is what loss of one-place trust involves. A confident style of anticipation gives way to pervasive and non-localized uncertainty and doubt, and a sense of danger predominates. We can thus see why someone might describe herself as living in a “different world.” Recalling the example of the musical note, how we experience what is present is shaped by what we anticipate. The point can be applied more specifically to the affective aspects of anticipation. When the realization of some indeterminate threat is anticipated, things can “look” foreboding. And when the overall style of anticipation takes this form, a sense of being confidently immersed in the world, “at home” in it, is lost. One feels “uprooted;” the world as a whole appears strangely and disturbingly different.

## INTERPERSONAL TRUST AS A SOURCE OF POSSIBILITY

Loss of confidence or “one-place trust” can take a number of forms. When faced with chronic illness, one’s bodily experience might involve a pervasive sense of what Carel (2013) calls “bodily doubt;” one ceases to habitually “trust” one’s bodily capacities and capabilities, and the default style of anticipation becomes that of anxious uncertainty. Alternatively, it could be focused upon the impersonal world; an environment that was previously taken to be dependable now seems dangerous and unpredictable. Or one might cease to trust one’s own abilities, perhaps even the reliability and coherence of one’s own thoughts. Hence it might be objected that the term “one-place trust” is misleading. Given that it can have different emphases, “trust in *p*,” “trust in *q*,” and so on, it actually falls under the category “two-place trust.” However, “having trust in the context of domain *p*” is to be distinguished from “trusting *p*.” The former is not a relation of trusting but a precondition for one or another broad kind of trust relation. So, although one-place trust has more circumscribed domains, it is not itself an attitude toward something.

The concern could also be raised that “trust” properly applies to the interpersonal domain and its extension to other forms of practical confidence is inappropriate. In response, it should be noted that the term “trust” is sometimes used in the more permissive way, and victims of trauma often refer to a wider-ranging “loss of trust in the world.” Even so, we will now suggest that having trust in other people has a kind of primacy over others forms of one-place trust. This is because its loss also entails a more general loss of confidence in oneself, one’s abilities, and one’s surroundings. Furthermore, where trust in some other domain is eroded, interpersonal trust more usually has an important role to play in its restoration. In the absence of interpersonal trust, other losses of trust are experienced as irrevocable rather than contingent.

Relations with other people serve to shape and re-shape our experiences and attitudes. Even mundane and short-lived interpersonal interactions can be self-affecting. Whether an expression, gesture, or comment is met with a smile or a dismissive sneer can have a subtle but wide-ranging effect on experience of oneself, the other person, and the surrounding environment. For this reason, Løgstrup (1956/1997, p. 18) proposes that all interpersonal relations involve unavoidable responsibility for others; we cannot interact with someone without somehow affecting his “world:”

By our very attitude to one another we help to shape one another’s world. By our attitude to the other person we help to determine the scope and hue of his or her world; we make it large or small, bright or drab, rich or dull, threatening, or secure. We help to shape his or her world not by theories and views but by our very attitude toward him or her. Here lies the unarticulated and one might say anonymous demand that we take care of the life which trust has placed in our hands.

According to Løgstrup, entering into any kind of interpersonal relationship involves a balance of trust and vulnerability. To relate to someone in a distinctively *personal* way is to be open to her potential influence on one’s world and thus vulnerable to harm. In doing so, one trusts the other person not to do harm – one’s life is “placed in her hands^11^.” Although that might sound rather dramatic, the relevant phenomenon is familiar and commonplace. Gallagher (2009) discusses how, as well as making sense of others through our interactions with them, we make sense of the world more generally. What we attend to is regulated by others, and there is empirical evidence suggesting that their presence alone serves to influence what we take to be salient, how we evaluate it, and how we respond to it. This applies from a very young age: “we learn to see things, and to see them as significant in practices of shared attention” (Gallagher, 2009, p. 303)^12^. What we take to be “salient” and “significant” is inseparable from what we anticipate – from what we think is likely to happen and how it matters. Hence interactions with others can shape the content, mode, and affective style of anticipation, in relation to however many features of the environment.

Given that what and how we anticipate is inextricable from our experience of what is present, our surroundings can “look” different depending on whether we are interacting with others and on what form the interaction takes. It is not so much a matter of what the other person says; she need not say anything. It is largely attributable to styles of interaction, to patterns of shared attention, to how gestures and expressions are elicited and followed up (although it can also involve the construction, elaboration, and revision of self-narratives). van den Berg (1972, p. 65) offers the following description:

We all know people in whose company we would prefer not to go shopping, not to visit a museum, not to look at a landscape, because we would like to keep these things undamaged. Just as we all know people in whose company it is pleasant to take a walk because the objects encountered come to no harm. These people we call friends, good companions, loved ones^13^.

Interactions with others can thus facilitate changes in perspective, which are often subtle but occasionally quite profound. After interacting for a prolonged period with a particular person, the world might seem strangely impoverished or, alternatively, alive with new possibilities. Hence the interpersonal serves to imbue things with a sense of contingency. The anticipation of entering into certain kinds of relation with others amounts to a sense that “this is not all the world has to offer,” an appreciation that there are other possibilities, however indeterminate those possibilities might be.

Traumatic events can elicit a shift in the overall style of interpersonal anticipation, in the balance between vulnerability and trust. What makes interpersonal trauma distinctive is the *subversion* of interpersonal trust that it involves. The other person recognizes one’s vulnerability and responds to it not with care but by deliberately inflicting harm. The aim of torture has been described as the complete psychological destruction of a person: “the torturer attempts to destroy a victim’s sense of being grounded in a family and society as a human being with dreams, hopes and aspirations for the future” (Istanbul Protocol, 1999, p. 45). It is a “calculated assault on human dignity,” more so than an attempt to extract information (Amnesty International, 1986, p. 172)^14^. The victim is confronted by a kind of interpersonal relation that exploits her vulnerability in an extreme way. Améry (1999, p. 29) describes how, when one is hurt, there is ordinarily an “expectation of help” from others, something that is engrained from early childhood. Hence torture involves a radical conflict with habitual styles of interpersonal anticipation. It is not just that others fail to offer help; they are themselves the agents of harm and there is nobody else to intervene on one’s behalf. Furthermore, many forms of torture involve taking familiar, homely items that would more usually be encountered in a confident, purposive way, and using them to cause harm. For instance, household utensils are sometimes used to inflict pain (Scarry, 1985, pp. 40–41). So it is not just that an interpersonal situation fails to offer what is habitually anticipated; it offers something utterly opposed to it^15^.

Such experiences can lead to a shift in the vulnerability–trust dynamic described by Løgstrup, whereby anticipation of harm becomes a salient aspect of interpersonal experience, shaping all interpersonal relations; one-place interpersonal trust is eroded or lost^16^. Exactly how this comes about is debatable (and our aim here is to describe the resulting experience rather than the mechanisms through which it arises). The victim might well form explicit judgments to the effect that “the interpersonal world is not as I took it to be,” which in turn influence her overall style of anticipation. However, it is unlikely that the change in anticipatory style occurs solely via this route. In many other contexts, conflicts between explicit evaluative judgments and anticipatory style are commonplace. For example, someone who is bitten by a dog may then experience dogs as menacing and unpredictable, despite “knowing full well” that the incident was anomalous. The point applies equally to the more profound and pervasive effects of interpersonal trauma.

Loss of interpersonal trust has wider effects. Without the assumption that others will offer assistance in moments of need, the impersonal environment also seems less safe. What was once anticipated with habitual confidence is now anticipated with uncertainty and dread:

When you think about everything on a deep level, […] you see that nothing in life follows any rules; you can’t rely on anything to be always true, ever. Nothing is constant and nothing is reliable, so nothing is “safe” to just simply believe in and be done with it. You are constantly looking at everything around you and re-assessing it, re-evaluating it as you get new information about it^17^.

The point also applies to trust in one’s own abilities, even to the reliability of one’s own judgments and thought processes. More usually, where there is doubt we turn to others for reassurance and support. Importantly, when trust in the impersonal environment or in one’s own abilities is damaged, trusting relations with others can help one to negotiate what has happened and move on. They establish a sense of contingency, opening up new possibilities, and facilitating new interpretations. When interpersonal trust is lost, the prospect of entering into an interpersonal process that might otherwise have enabled a shift in anticipatory style is lost along with it. As Laub (2001, p. xv) observes,

…….the survivor of torture feels completely alone. He – or she – no longer believes in the very possibility of human connection; he envisages no one who will be present to him and for him if he returns in his mind to the places of horror, humiliation, and grief from which he barely emerged and which continue to haunt him.

Consequently, one’s predicament is not experienced as a contingent one; the world no longer offers anything else. The resultant experience can also involve a sense of revelation, as a confidence so deep-rooted that it was never questioned reveals itself as utterly misplaced^18^. This further exacerbates the experience of alienation from others. Even when someone else is not encountered as threatening, he resides somewhere else, in a place where innocence remains and people go about their business in a confident – albeit naïve – way.

## LOSS OF A MEANINGFUL FUTURE

Projects, cares, and concerns are sustained interpersonally. Almost all goal-directed activities implicate other people in some way – one is asked to do things by others and for others, and one does so in collaboration with others. The integrity of one’s projects therefore depends on the integrity of those relations. Where there is pervasive uncertainty, where others cease to be dependable, where the world is unsafe and one’s own abilities are in doubt, projects collapse. It is not just that the person lacks something that is presupposed by the possibility of a *specific project*. What is missing is something that the intelligibility of *projects in general* depends upon. One finds oneself in a world from which the possibility of meaningful, progressive, goal-directed activity is absent. Other kinds of concern are affected in other ways. For instance, care for certain other people may endure, but a pervasive sense of the world as unsafe and unpredictable renders it fragile and vulnerable. One inhabits a place that is inhospitable to human relationships. Interpersonal care is thus coupled with the anticipation of impending and inevitable loss, with dread, and anticipatory grief.

Such an experience has a profound effect upon one’s beliefs. Beliefs involving positive evaluations of future events in relation to ongoing projects cease to be intelligible, given that such projects have collapsed. In addition, one ceases to anticipate the future with habitual confidence and no longer takes it to be the case that “*p* will happen” or “*q* will happen;” everything seems less certain. There is also a more widespread effect upon one’s beliefs. Various factual beliefs that were once asserted with confidence may now seem hollow, irrelevant, and alien, given that their relevance and significance depended upon projects that have been lost. More generally, there is a change in the *way* one believes; things are no longer taken to “be the case” with a sense of confident certainty. That kind of certainty is gone from the world, and nothing stands firm in the way it once did. Furthermore, other people cannot be relied upon for testimony and correction of errors, and one’s own intellectual abilities are experienced as all the more suspect without their reassurance.

A person’s philosophical beliefs are not insulated from these phenomenological changes. Some of them, perhaps even the vast majority, presuppose a confidence that is “shattered” in trauma. When the confidence that one’s philosophical projects depend upon is lost, one can still utter various propositions and argue over them, but the activity takes on an air of absurdity. The seeming irrelevance of much philosophical discourse following traumatic experience is noted by Brison (2002, p. x), herself an academic philosopher: “when I was confronted with something strange and paradoxical, philosophy was of no use in making me feel at home in the world^19^.” As both Husserl (1948/1973) and Wittgenstein (1975) suggest (in different ways, admittedly), the possibility of believing that *p* or believing that not *p* depends upon a different kind of confidence. We suggest that, when that confidence is disturbed, one does not believe in quite the same way anymore. A form of enquiry concerned with whether we should believe that *p* or that *q* is irrelevant when something presupposed by that kind of believing is itself in jeopardy^20^.

A change in the style of anticipation and conviction, of the kind that renders projects unsustainable, also amounts to a change in the short-term and longer-term sense of time. In the case of short-term time, there is a shift in the structure of protention. One’s style of anticipation is bereft of certain kinds of possibility, such as that of something happening that matters in a good way, or – more specifically – something that builds upon what one has achieved up to now. Hence there is a change in the experience of what we might call temporal “flow” or “passage,” which no longer involves the anticipation and actualization of certain meaningful kinds of possibility. With this, the person is no longer “moving forward,” “heading somewhere,” and so there is also an altered sense of temporal direction. The longer-term sense of time is also very different. When the person looks ahead, the future lacks structure; it is not ordered in terms of meaningful projects, and so a coherent sense of long-term duration is absent. Hence the all-enveloping dread she feels before some inchoate threat is not situated in relation to a wider pattern of meaningful temporal events. There is nothing meaningful between now and its actualization, and so it seems imminent. A loss of interpersonal trust that is central to this form of experience is also what sets it in stone. Without the possibility of entering into trusting relations with others, the predicament seems unchangeable. There is no access to the process that might otherwise reveal its contingency and allow her to move beyond it. The person is isolated from others in a way that is incompatible with “moving forward in time;” her life story has been cut short.

This experience is not just future-oriented; it also affects how one’s past is experienced. Past activities and events are significant insofar as they relate to where one is going, insofar as they are further developed, compensated for, or left behind. The past is thus constantly renegotiated, reinterpreted:

…the future is the site of both anticipation and the unexpected, planning and the changing of plans. This predominant orientation toward a changing future also means a fluid or unfixed past, because the past is continually being reassessed as one moves into the future (Havens, 1986, p. 21).

When the possibility of moving forward in a purposive, progressive, structured way is absent, so is that of reinterpreting one’s past. So we can also see why traumatic memories might be experienced as vivid, intrusive flashbacks, why they are “relived” more so than “recalled” (e.g., Hunt, 2010, p. 70). The traumatic event is not contextualized or re-interpreted in relation to where one is heading, because the kind of trust required to move on has been lost. This is not to suggest that a traumatic memory endures as a wholly unadulterated record of how the traumatic event was experienced at the time. Our point is that it is not contextualized in the *way* that remembered events more usually are. This may also account for the intrusive nature of traumatic memories. As they are not integrated into a coherent life story, the person does not first recall another, related part of the story and – in the process – anticipate their coming. They are “triggered” or “cued” in a different manner and arise without prior context. To speculate further, difficulties in recalling traumatic memories may equally be attributable to this lack of contextualization. That they are not integrated into a structured life narrative makes them harder to actively recall or – alternatively – easier to avoid^21^. Other memories of events prior to the trauma are interpreted and re-interpreted, but only up to that point. A life story therefore seems complete, cut short by something that the person continues to confront but cannot negotiate^22^.

Hence a sense of foreshortened future is not a judgment to the effect that the remainder of one’s life will be short and that one has little or nothing to look forward to. It is a change in how time is experienced: an orientation toward the future that is inseparable from one’s experience of past and present, and also from the short- and long-term “passage” of time, is altered. It is not just that one will no longer get married, have children or have a successful career. One confronts a world that is incompatible with the possibility of an open and progressive life story^23^. And so traumatized people sometimes describe themselves as having died or say that a part of them has died: “when trust is lost, traumatized people feel that they belong more to the dead than to the living” (Herman, 1992/1997, p. 52).

This can be conceived of in terms of what Heidegger (1927/1962) calls “being-toward-death.” Joseph Rouse, in a conference paper on John Haugeland’s interpretation of Heidegger, develops the point that being-toward-death or “existential death” involves anticipating something that is distinct from biological death^24^. Existential death, Rouse explains, “is not an actual event, but a comportment toward the ever-impending possibility of [our] own impossibility.” In other words, it is the recognition of the potential unintelligibility of something that the intelligibility of all our projects, cares and commitments depend upon, of something that many of us are oblivious to in the course of everyday life. The most extreme form of traumatic experience can be thought of as the *actualization* of existential death within a continuing conscious life. One’s life is experienced as having ended because its conditions of intelligibility have collapsed. This, we suggest, is how remarks about having died should sometimes be understood: “for months after my assault, I had to stop myself saying (what seemed accurate at the time), ‘I was murdered in France last summer’ ” (Brison, 2002, p. xi)^25^. There are two distinct ways in which the actualization of “existential death” might be construed. Our activities are intelligible within wider-reaching projects and so the potential unintelligibility of those activities is intelligible in the guise of losing the relevant projects. Indeed, human lives are peppered with project-deaths; a project can collapse for a variety of reasons, rendering some but not all of one’s activities unintelligible, after which one is often able to move on. However, existential death can – and should – be construed more strongly in terms of the loss of something that is presupposed by the intelligibility of *any* project, and this is what is occurs in some experiences of interpersonal trauma. A life story is over not because some set of projects that are fundamental to one’s life has been lost but because the possibility of engaging in any such project has been lost along with them. This, we stress, is an *extreme* form of the experience, and a trusting style of anticipation can be eroded to varying degrees.

## CONCLUSION

We concede that experiences of trauma are heterogeneous and that diagnoses such as PTSD are compatible with a range of subtly different predicaments. Even so, our account suggests a phenomenological unity to various symptoms that might otherwise be regarded as closely related but distinct. The DSM-IV-TR description of PTSD includes the following symptoms (along with several others): “impaired affect modulation,” “feelings of ineffectiveness, shame, despair, or hopelessness,” “feeling permanently damaged,” “a loss of previously sustained beliefs,” “social withdrawal,” “feeling constantly threatened,” “impaired relationships with others,” and “a change from the individual’s previous personality characteristics.” Feeling threatened, socially withdrawn, and unable to relate to others can all be construed in terms of losing interpersonal trust. The same applies to impaired affect regulation: the person cannot participate in interpersonal relations of a kind that more usually serve to regulate experience, thought, and activity. We have also suggested that loss of trust has a profound effect on what a person believes and the *way* in which she believes. In addition, loss of trust amounts to a sense that one’s life story has been cut short and therefore to a feeling of being irreparably “damaged.” There are perhaps two aspects to feeling ineffective: (i) loss of interpersonal trust disposes one to lose trust in one’s own abilities; (ii) in a world where meaningful action is impossible, one is unable to do anything of consequence, something that might be expressed in terms of ineffectiveness. And all of this surely amounts to a substantial “personality change.” Hence empathizing with extreme traumatic experiences (where “empathy” is understood in a non-committal way as coming to understand an experience, rather than as trying to somehow “experience it in a first-person way”) involves coming to appreciate a profound and unitary phenomenological change, one that does not concern however many experiences of *p* and attitudes toward *q* but an overall style of experience that they presuppose.

It is also important to emphasize the interpersonal, social, and cultural aspects of trauma. We have argued that traumatic experience is essentially a way of relating to people in general. How specific others and others in general respond to one’s experience may serve to mitigate or exacerbate the loss of trust that is so central to it. Turning first of all to exacerbation, our approach can contribute to an understanding of the link between trauma and psychosis (Istanbul Protocol, 1999, p. 47). We have emphasized the indispensable regulatory roles that other people play, how our relations with them shape our experiences, thoughts, projects, goals, and life stories. Given this, the development of psychosis following trauma is not to be construed solely or even principally in terms of processes that are internal to an individual. Our beliefs are shaped by our interactions with others, who provide reassurance and correction. So a pervasive estrangement from other people affects the *ways* in which beliefs are formed, maintained, and revised. This kind of view is hinted at but not fully developed by Jaspers (1963, p. 104), who observes that our beliefs are integrated into a public world, to which the status “incorrigible” usually attaches. This sense of being embedded in a dependable, predictable, public world can be altered by traumatic events, such that a certain kind of conviction, a way of believing, is altered. As Jaspers adds, “socially accepted reality totters, people become adrift.” The point can be extended from belief-formation to how we experience our surroundings, even to our ability to distinguish what is physically present from what is not. Guenther (2013, p. 146) offers the following remarks, in relation to extreme forms of social privation that prisoners in long-term solitary confinement are subjected to:

We rely on a network of others, not just to survive or to keep ourselves entertained but also to support our capacity to make sense of the world, to distinguish between reality and illusion, and even to tell where our own bodily existence begins and ends.

It is arguable that post-traumatic estrangement from others and complete loss of felt interpersonal connection can similarly serve to erode one’s sense of what is real, and of the boundaries between self, other people, and the surrounding world, in a way that renders one susceptible to symptoms such as hallucinations and delusions. We can add to this that loss of trust in the world involves a pronounced and widespread sense of unpredictability. This unpredictability, further cultivated by increasing social isolation, may render a person susceptible to further disturbances in the style of anticipation^26^. Hence it would be interesting to explore how an all-enveloping loss of trust relates to and perhaps contributes to “salience dysregulation” of the kind linked to psychosis, where things appear significant in anomalous and unstructured ways (Kapur, 2003; Kapur et al., 2005). Of course, not all severely traumatized people go on to develop psychosis, and different trajectories as well as prior vulnerabilities need to be considered (Herman, 1992/1997). Even so, where psychosis does arise in the context of trauma, our account suggests that an interpersonal, rather than intrapersonal, process is at work. The point applies equally to other trajectories that traumatic experience might follow.

With regard to mitigation, successful therapy can involve changing the person’s sense of what others have to offer, in a way that facilitates re-integration into the public world. Herman (1992/1997) describes three broad stages of recovery: a localized sense of safety is first nurtured, after which the person can attempt to construct a narrative around what has happened, and finally there is reengagement with communal life. What we have said is consistent with this general approach. To begin with, certain possibilities may not even make sense to the person. So encouraging her to do various things, adopt certain attitudes, or change her perspective on life is analogous to encouraging her to swim to safety when she finds herself stranded on a desert planet with no prospect of escape. Given that trust is a precondition for even entertaining certain possibilities, a degree of trust first needs to be restored^27^. This is not to suggest that a victim of interpersonal trauma can ultimately recover the same style of unreflective trust that previously permeated her world. But she can come to relate to others and to the world more generally in a way that is compatible with moving forward into an open future^28^.

## Conflict of Interest Statement

The authors declare that the research was conducted in the absence of any commercial or financial relationships that could be construed as a potential conflict of interest.

## Footnotes

1 This can involve punishment for something the victim or someone else has done, where the aim is to punish the victim and/or punish somebody else who cares about the victim.

2 See, for example, Peters (1996) for several different definitions of torture.

3 As suggested by ICD-10, in such cases it is “unnecessary to consider personal vulnerabilities in order to explain its profound effect on the personality” (1992, p. 209).

4 There are further complications in cases such as extended solitary confinement, where harm is inflicted by others in the relevant way but chronic sensory and social deprivation have additional effects on temporal experience. For recent discussions, see Grassian (2006) and Guenther (2013).

5 For example, Jones (2004, p. 6) offers the following analysis of three-place trust: “trust is accepted vulnerability to another person’s power over something that one cares about, where (1) the truster forgoes searching (at the time) for ways to reduce such vulnerability, and (2) the truster maintains normative expectations of the one-trusted that they do not use that power to harm what is entrusted.”

6 See also Baier (1986), Stolorow (2007, 2011), and Bernstein (2011) for complementary descriptions of what we have called “one-place trust.”

7 Experiences of depression are often described in these terms too. See Ratcliffe (in press) for a detailed discussion.

8 Alternatively, belief could be distinguished from an affective, practical responsiveness that usually accompanies it. For example, Gendler (2008) distinguishes “belief” from what she calls “alief.” Those who accept such a distinction can say that *x* believes that *p* and that, following the traumatic event, *x* also comes to alieve that *p*.

9 As Corbí (2012, p. 55) observes, a “certain kind of awareness of some facts, deeper than mere knowledge of them, seems to be required to account for the conditions under which our confidence in the world may be lost.”

10 There are interesting similarities between Husserl’s account of habitual certainty, as something that our various beliefs about what is and is not the case presuppose, and some of the later Wittgenstein’s remarks concerning “hinge propositions.” According to Wittgenstein, when we take something to be the case or otherwise, we presuppose a different kind of certainty. Hinge propositions, he says, are not “propositions” of a kind that we could take to be true or false; they are the “ground” for judgments of truth and falsehood (Wittgenstein, 1975, p. 27). At times, Wittgenstein (1975, p.22, 66) describes this ground as a form of “trust” and emphasizes the practical nature of the relevant kind of certainty. See Moyal-Sharrock (2005) for a discussion of the role of trust in Wittgenstein’s *On Certainty*.

11 See Bernstein (2011) for an account along similar lines.

12 See also Seemann (2009) for a complementary discussion of what he calls “basic” interpersonal trust and the nature of joint agency.

13 Similar descriptions are offered by various others. See, for example, Gendlin (1978/2003, p. 115).

14 See also Scarry (1985) for a sustained argument to the effect that the aim of torture is not to interrogate but to destroy the person. For a comprehensive and convincing critique of the view that torture is an effective means of interrogation, see Costanzo and Gerrity (2009).

15 Extended periods of solitary confinement can similarly involve subversion of a previously habitual anticipatory style. The person is deliberately starved of kinds of interpersonal relation that were previously taken for granted, and the only kind of social relation that remains is one of powerlessness in the face of an individual or collective that deliberately inflicts harm (Guenther, 2013).

16 It seems plausible to suggest that this outcome is more likely in certain scenarios than others. For instance, where the harm is inflicted and/or endorsed by a group rather than an individual, perhaps even state-sanctioned, this lends itself to the view that people in general are suspect. It is also important to recognize the role of third-parties. Corbí (2012) emphasizes the “secondary expectation” that, even when I am harmed by someone, others will come to my aid. Their failure to do so, their refusal to engage with what has happened and their even siding with the torturer in order to maintain their own sense of confidence in others can all exacerbate a loss of trust.

17 This testimony is taken from a questionnaire study, which was conducted as part of AHRC- and DG-funded project “Emotional Experience in Depression” and ethically approved by the Durham University Philosophy Department Research Committee. This respondent (no. 179) had diagnoses of depression and PTSD.

18 Stolorow (2007, p. 16) describes this as a “catastrophic loss of innocence that permanently alters one’s sense of being-in-the-world.”

19 See also Corbí (2012, p. 45).

20 In a stronger statement, Améry (1999, p. 19) contrasts the reality of life in Auschwitz with the attempts by some of those in the camp to sustain intellectual debates: “to reach out beyond concrete reality with words became before our very eyes a game that was not only worthless and an impermissible luxury but also mocking and evil.”

21 There are potential implications here for our understanding of dissociation in trauma. The term “dissociation” is used to refer to various different kinds of experience and to different aspects of an experience. For example, it sometimes refers to a sense of “derealization” and/or a feeling of detachment from one’s own body, both of which are integral to the loss of trust that we have described. The person feels disconnected from the world, as it no longer appears practically significant in the way it once did – she is not quite “there.” And, insofar as she lacks certain feelings of anticipation and associated bodily dispositions, insofar as she is no longer practically and habitually immersed in situations, her body may feel strangely inanimate and alien too. However, where “dissociation” arises in relation to memory, a different account is required. What we have described in this section could be regarded as a form of dissociation – decontextualized memories are experienced as both estranged and estranging, and the person may also have trouble accessing them. But a different kind of “dissociation” is involved when the person manages to retain trust in the world and move forward by *not* confronting or engaging in any way with what has happened. The resultant experience would be quite unlike what we have focused upon in this paper, but the traumatic memory would similarly remain detached from a life story. It is debatable whether and when this latter kind of dissociation is adaptive, protecting one from a collapse of trust of the kind we have described, and when it is itself part of the problem. That these two different kinds of “dissociation” may be implicated in trauma complicates discussion of the link between dissociation and “reality discrimination” (see, e.g., Varese et al., 2012a). Some forms of post-traumatic dissociation may be symptomatic of finding oneself in a “different world” while others involve the preservation of one’s world.

22 Stolorow (2011, p. 55) describes such an experience as follows: “experiences of emotional trauma become freeze-framed into an eternal present in which one remains forever trapped, or to which one is condemned to be perpetually returned.[….] In the region of trauma, all duration or stretching along collapses; past becomes present, and future loses all meaning other than endless repetition.”

23 The changes in temporal experience that we have described overlap with how some people with depression diagnoses experience time. In both cases, there is a closed past, a future that offers only threat, and no sense of meaningful transition between past, present or future (Ratcliffe, 2012). Something that distinguishes an experience of trauma is the sense that a past which *was* meaningful and perhaps continues to be remembered as meaningful has been *cut short* by some event. Along with this, one “relives” the event, confronts something that cannot be negotiated. However, the distinction is difficult to draw in cases of sustained trauma, which often originate in childhood, where the person either never gained trust in the first place or lost it in a way that cannot be pinned down to a particular event.

24 Rouse’s paper, entitled “Love and Death,” was presented at “Mind, Meaning and Understanding: A Conference in Honor of John Haugeland” (University of Chicago, Chicago, May 2010) and is available at: https://wesfiles.wesleyan.edu/home/jrouse/Love%26Death.doc (accessed 4th April 2014).

25 “Existential death,” as we have described it, relates closely to what others have referred to as “social death” or “social murder,” construed as a radical form of social disenfranchisement that is incompatible with sustaining a meaningful human life (Bernstein, 2011; Guenther, 2013).

26 There is increasing recognition that affective disturbances are implicated in psychosis (e.g., Smith et al., 2006). See Morrison et al., 2003 for the view that there is considerable overlap between the symptoms of psychosis and trauma, that they interact causally in a range of ways, and that interpersonal processes have a role to play. See also Earnshaw (2011) for the view that progressive erosion of trust is involved in the development of psychosis. See Varese et al. (2012b) for a meta-analysis pointing to the conclusion that traumatic events during childhood significantly increase the risk of psychosis. There is also a more specific link between childhood trauma and “hallucination-proneness” (Varese et al., 2012a). As stressed earlier, we have not addressed childhood trauma in this paper. The emphasis on trust is likely to prove equally central here, but an important difference is that trust in others, and in the world more generally, is disrupted during its formation, rather than later “shattered.” This requires further discussion, as does the issue of how childhood trauma can render one more vulnerable to further loss of trust in later life. Childhood trauma has been linked to a range of different psychiatric diagnoses in adulthood (Read et al., 2005).

27 See also Janoff-Bulman (1992) for an emphasis on the substantial role that social support has to play and for distinctions between various complementary types of social support.

28 The phenomenological study of trauma also has potential implications for philosophical approaches to temporal experience. Philosophers often characterize the experience of “temporal flow” or “passage” in a cursory way, after which discussion turns to the issue of whether any mind-independent temporal properties correspond to an experience of flow. For example, Paul (2010) refers simply to an experience of “newness” and “passage” and concludes that our brains create the illusion of “flow.” (See Hoerl, 2014, for a good discussion of this and similar approaches, and of problems involved in even making sense of the view that we have an illusory experience of passage.) Before drawing such conclusions, it is advisable to formulate a sufficiently detailed and nuanced account of one’s subject matter. Along with various others, Paul (2010, p. 346) assumes that an “experience of passage” is to be identified with one of “change.” However, consideration of temporal experience in trauma indicates that matters are not so clear. The traumatized person continues to register “change.” Even so, her sense of the transition between past, present and future is profoundly altered and she no longer experiences herself as “moving forward” in time, as her experience of temporal transition is no longer one of meaningful transition. The nature of one’s temporal experience is inextricable from one’s style of anticipation. Furthermore, it is intersubjective in structure, involving ways of relating to other people that are ordinarily so entrenched we seldom notice them. It is debatable what the implications of this are for the philosophy of time. But it does point to the view that philosophical discussions often operate with a conception of their subject matter that is cursory and unclear. In order to sensibly address the issue of whether some *x* is or is not illusory, we need an adequate understanding of what *x* is and what *x* is not. The acknowledgment that there are different *ways of being in time*, involving different experiences of transition, complicates matters.
